# A Minor Innovation in Constructing a Small Bowel Stoma in Neonates with Small Bowel Atresia to Reduce the Morbidity

**DOI:** 10.21699/jns.v5i4.474

**Published:** 2016-10-10

**Authors:** Naeem Khan, Saba Bakht, Nadia Zaheer

**Affiliations:** 1 KRL General Hospital, G-9/1, Mauve Area, Islamabad; 2 IBM S.P.A Italia, Islamabad, Pakistan

**Keywords:** Intestinal atresia, Chimney, New modification, Morbidity

## Abstract

Background: Intestinal atresia has still significant morbidity in developing countries. Stomas are now not recommended in every case of intestinal atresia; primary anastomosis is the goal of surgery after resection of dilated adynamic gut. A new type of stoma formation along with primary anastomosis is being presented here.

Materials and Methods: This report is based on our experience of many cases with this technique in last 12 years but all the details and long follow-up of each case is not available. However the method of surgical procedure, progress, complications, and advantages encountered have been highlighted.

Results: Presently we have data of 7 patients; others are lost to follow up. Three had died with other associated problems, namely one with multiple atresias, two with septic shock and prematurity. Two stomas did not require formal closure because stoma shriveled and disappeared. Two other stomas had grown very long like a diverticulum when these were closed after 5 and 8 months.

Conclusion: This technique is another attempt to decrease morbidity of patients of intestinal atresia especially in those cases where short bowel syndrome is feared after resection of proximal dilated gut.

## INTRODUCTION

Stoma of small bowel and large bowel are constructed for different reasons, ranging from decompression and bypass to relieve a distal obstruction, to protect distal anastomosis, for enteral feeding, or for irrigating the bowel to give a anti grade enema [1-4]. Unfortunately, it is a procedure which has many complications like bleeding, prolapse, ulceration, stenosis adhesions and obstruction. It causes revulsion to parents and onlookers and is difﬁcult to manage especially for the mother who has to look after other children also. In proximal stomas patients often become anemic, develop frequent diarrhea with ﬂuid and electrolyte losses, requiring frequent hospitalization. Because of these complications different innovative techniques have been designed to prevent or at least reduce these complications and associated morbidity. In the background of these problems we have devised a procedure using a segment of dilated small bowel which would otherwise have been discarded during the tailoring of the dilated gut segment. Various steps and precautions of the procedure are described. Advantages and beneﬁts as well as observed complications have been described.

## MATERIALS AND METHODS

This report is based on our experience with this technique in last 12 years but all the details and long follow-up of each case is not available. However the method of surgical procedure, progress, complications, and advantages encountered have been highlighted. Pictorial record presented here depicts well the objectives of our presentation.

Technique: In the past we used to resect the dilated segment of gut but now believe that where possible this segment should not be sacriﬁced because this part of intestine may be important part of gut where essential elements and ﬂuid and electrolytes may be absorbed and exchanged. Auto stapler liner cutter is employed in the long axis of the proximal dilated blind ending gut which divides the bowel into two conduits (V-shaped two bowel channels) (Fig.1,2). We always reinforce the suture line with interrupted 5 zero Vicryl/PDS on a round body needle. Special care is taken to close the "V" tip of the divided stapled gut. We always try to avoid holding the gut with instruments and unnecessary handling especially the antimesenteric segment which is relatively ischemic, instead we use stay sutures to move and retract the segment. The mesenteric bowel conduit is used to anastomose with the distal gut to restore bowel continuity (Fig.3). Antimesenteric conduit is used to form an temporary enterostomy Distal half of the conduit which is mostly dusky is trimmed off leaving only about 2 to 2.5 cm for bringing it out as stoma, taking special care not to twist the conduit and at least partially draw it out through extra peritoneal rout (Fig.3,4). Since it is a narrow conduit, constructed stoma is also like a tip of a little ﬁnger and can be brought out anywhere from the primary wound. Care is taken that the stoma is not under tension and is not twisted only four stitches are used to anchor the stoma. Stoma can also be brought out through a separate incision. A well lubricated size 6Fr nasogastric tube is placed through the stoma for decompression to protect the anastomosis, and later it can also be used for contrast studies and for additional feeding. Wound is then closed in layers. Postoperative care is given in usual routine.

**Figure:**
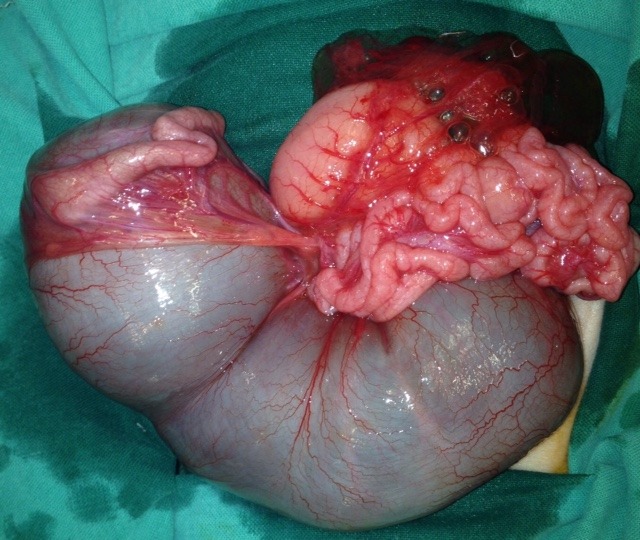
Figure 1: Jejunal Atresia, ideal for such technique.

**Figure:**
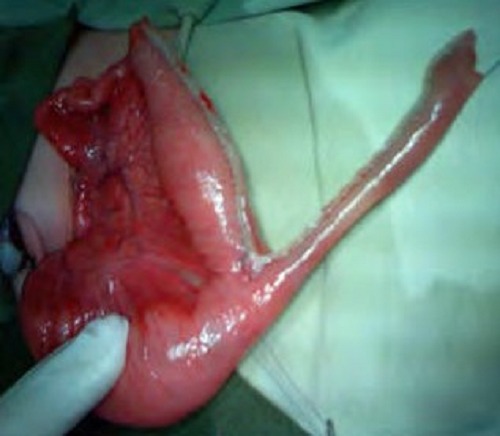
Figure 2: GIA auto stapler linear cutter is used to divide the proximal dilated portion of an intestinal atresia which divided it into V-shaped two conduits. The entire suture line is reinforced with additional layer of manual suturing with Vicryl/PDS 5/0.

**Figure:**
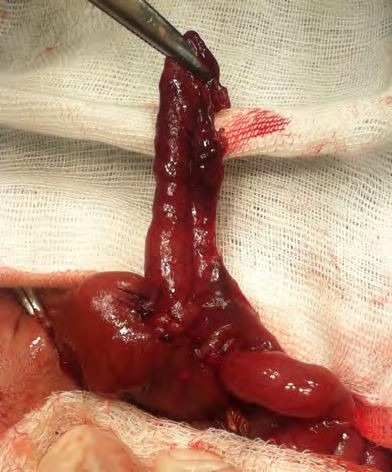
Figure 3: Showing the mesenteric conduit has been used to restore bowel continuity with the distal part of bowel atresia. The antimesenteric part is ready to bring out as enterostomy. The distal dusky portion will be excised leaving behind only 2.3cm of conduit.

**Figure:**
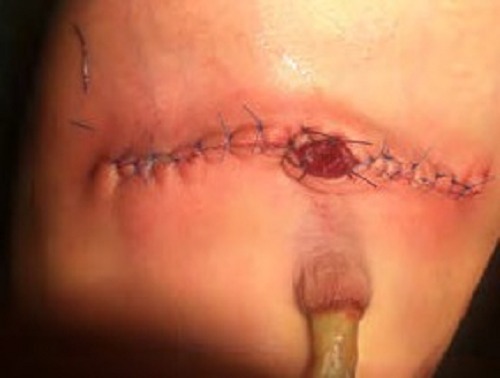
Figure 4: Antimesenteric bowel conduit after stoma formation.

## RESULTS

Many patients had undergone this operation for more than a decade however their record neither be retrieved and nor the patients presented to us for follow-up. Presently we have data of 7 patients; others are lost to follow up. Three had died with other associated problems, namely one with multiple atresias, two with septic shock and prematurity. Two stomas did not require formal closure because stoma shriveled and disappeared. Two other stomas had grown very long like a diverticulum when these were closed after 5 and 8 months (Fig.5,6). These stomas were totally asymptomatic and almost with no discharge of intestinal contents. Closure of these stomas is similar to any ostomy closure. Care is taken while closing such stomas not to narrow bowel lumen. This reversal procedure is performed at the age of three to four months.

**Figure:**
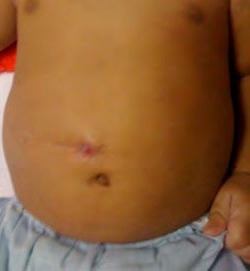
Figure 5: Appearance of stoma after few months of operation.

**Figure:**
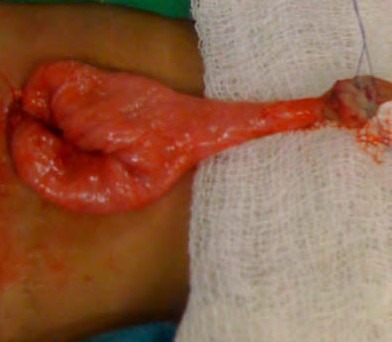
Figure 6: Sometimes conduit undergoes excessive growth in length. On the other hand, if the blood supply is inadequate it may shrivel and get stenosed.

## DISCUSSION

Use of stoma is what we consider a necessary evil, is full of distress for the parents and pain and for the baby, management is not an easy task, especially where the parents are poor and uneducated. Diarrhea and ﬂuid/electrolytes losses continue to cause skin excoriations, ulcerations, bleeding, anemia, repeated hospital admissions due to severe dehydration. It is due to these problems different people have tried different innovative methods to reduce these complications. With this method we have also tried to reduce the ﬂuid losses to the minimum and minimize other complications and feel we have reasonably succeeded, at the same time the purpose of decompression and protection of distal anastomosis is adequately served.

## CONCLUSION

The salient features of making this conduit is that the antimesenteric segment of tapered bowel is not discarded and proximal part of this is employed as a stoma conduit for protection of distal anastomosis, temporary decompression of proximal bowel, and performing a contrast study in addition to giving feeds through it.

## Footnotes

**Source of Support:** None

**Conflict of Interest:** None
